# Stromal Myofibroblasts Are Associated with Poor Prognosis in Solid Cancers: A Meta-Analysis of Published Studies

**DOI:** 10.1371/journal.pone.0159947

**Published:** 2016-07-26

**Authors:** Liu Liu, Lin Liu, Han Hui Yao, Zhi Qiang Zhu, Zhong Liang Ning, Qiang Huang

**Affiliations:** 1 Department of General Surgery, An Hui Provincial Hospital affiliated with the An Hui Medical University, He Fei, An Hui Province, China; 2 Department of Anesthesiology, An Hui Provincial Hospital affiliated with the An Hui Medical University, He Fei, An Hui Province, China; Seoul National University, REPUBLIC OF KOREA

## Abstract

**Objective:**

Published studies have evaluated the impact of stromal myofibroblasts on prognosis in solid cancers. However, the results of these studies remain controversial. We therefore performed a meta-analysis to address this issue.

**Methods:**

The PubMed, ISI Web of Science and Embase databases were searched through November 30^th^, 2015 by two investigators, and a total of 17 studies that contained 2606 patients were included. Stromal myofibroblasts were quantified in solid cancers using α-smooth muscle actin staining. Pooled Odds Ratio with 95% Confidence Intervals were calculated, and publication bias was analyzed.

**Results:**

The results of this study suggest that in solid cancers, a high density of stromal myofibroblasts is significantly associated with poor 3- and 5-year overall survival (pooled odds ratio (95% confidence interval): 1.33 (1.10–1.60) for 3-year overall survival and 1.68 (1.22–2.32) for 5-year overall survival). In addition, a high density of stromal myofibroblasts also predicted poor 3- and 5-year disease-free survival (1.30 (1.05–1.60) for 3-year disease-free survival and 1.36 (1.01–1.83) for 5-year disease-free survival). However, stromal myofibroblasts were not associated with 3- and 5-year cancer-specific survival. No publication bias was found for all analyses.

**Conclusions:**

The results of this study suggest that a high density of stromal myofibroblasts is associated with poor survival in solid cancers. More studies were required to investigate the prognostic value of stromal myofibroblasts in different types of solid cancers.

## Introduction

Solid cancers, such as gastric, liver and lung cancers, have become the leading cause of death around the world [1,2]. Despite the enormous advances that have been made in surgical and radiochemotherapeutic treatments, the prognosis for solid cancer patients remains unfavorable [2]. It has been acknowledged that local recurrence and distant metastasis are the main reasons for the poor prognosis in these patients [3,4]. Therefore, developing methods to efficiently identify patients who are at high risk of a poor prognosis is critical.

In solid cancers, the prognosis is determined not only by the oncological characteristics of the cancer cells but also by the micro-environment of the tumor [5,6]. The tumor micro-environment provides essential nutrients for tumor growth, inhibits immune surveillance against cancer cells and induces cancer cells to take on a more malignant phenotype [5,6]. Targeting the tumor stroma has therefore shown great potential as a cancer treatment [7,8].

The TNM staging system has been widely used to divide solid cancers into early or advanced stages. In this system, the staging is determined by the depth of cancer invasion, regional lymph node involvement and distant metastasis [9]. The TNM staging system undoubtedly provides valuable prognostic information for solid tumors and is also useful for determining the optimal treatment strategy to implement after surgery. However, it has been reported that cancers with the same TNM stage can have distinct prognoses. Thus, the TNM staging system is incompletely adequate for staging cancers [10]. The TNM staging system is partially limited by the fact that it relies on determinations related to the biological characteristics of the tumor cells but ignores the tumor environment. Hence, it is important that we identify supplementary markers that are specific to the cancer environment to assist in predicting prognoses in solid cancers [11].

The cancer stroma is a highly heterogeneous structure that is composed of activated fibroblasts, fibroblast-produced extracellular matrix, inflammatory cells (such as macrophages) and capillaries. Cancer-associated fibroblasts are the main component of the tumor stroma, and these have been paid a large amount of attention because of the prominent roles they play in cancer development, progression and metastasis [5,12]. Cancer-associated fibroblasts are high heterogeneous, and they can originate from residual fibroblasts, vascular smooth muscle cells, endothelial cells and pericytes. Fibroblasts in the tumor stroma transdifferentiate into myofibroblasts that exclusively express α-smooth muscle actin. The myofibroblasts in a cancer stroma represent a subgroup of cancer-associated fibroblasts [13]. It is widely accepted that myofibroblasts have a significant impact on wound healing because they affect extracellular matrix remodeling, secrete growth factors and enhance angiogenesis. Cancer lesions are similar to “unhealed wounds” [14], and myofibroblasts in the cancer stroma express a variety of growth factors and inflammatory chemokines that are involved in the remodeling of the tumor stroma, the regulation of the motility of cancer cells and the induction of tumor cells toward phenotypes that are more resistant to chemotherapy [15].

Because myofibroblasts play significant roles in the cancer stroma and during cancer progression and metastasis, they are viewed as good predictors of cancer prognosis and therefore potential targets for cancer treatments [16–18]. Many published studies have evaluated the impact of stromal myofibroblasts on prognoses in solid cancers by using α-smooth muscle actin as a molecular marker. Some studies have reported that stromal myofibroblasts are associated with a poor prognosis in solid cancers [19,20]. However, other reports have not come to the same conclusions [21,22]. The prognostic value of stromal myofibroblasts in solid cancers therefore remains unclear. Hence, in this study, we searched for relevant published studies and performed a meta-analysis to address this issue.

## Materials and Methods

This meta-analysis was conducted according to the guidelines of the preferred reporting items for systematic reviews and meta-analyses statement (S1 PRISMA Checklist) [23].

### Literature searches

Two investigators (Liu Liu and Lin Liu) searched the PubMed, ISI Web of Science and Embase electronic databases through November 30^th^, 2015. The search terms were: (myofibroblast OR (alpha smooth muscle actin)) AND (cancer OR tumor OR carcinoma) AND (survival OR prognosis OR prognostic). The reference lists of included studies and relevant reviews that were published during the past five years were screened to identify additional publications.

### Literature selection

The inclusion criteria for this study were: (1) the study investigated the association between stromal myofibroblasts that were positive for α-smooth muscle actin staining and prognoses in solid cancers, such as gastric, colorectal and breast cancer; (2) the expression of α-smooth muscle actin was assessed in the tumor stroma using immunohistochemistry; (3) the article provided sufficient data to obtain an estimated odds risk and a 95% confidence interval; and (4) the article was written in English.

The exclusion criteria applied in this study were: (1) the article reported duplicated data (if two or more articles used the same data, the latest published article was included); (2) the expression of stromal α-smooth muscle actin was determined using other methods, such as reverse transcription-polymerase chain reaction; (3) the article was reported in a non-English language; (4) the report lacked enough information for a combined analysis; and (5) the report was a review, comment or letter.

### Endpoints of interest

The primary endpoints in this meta-analysis were 3- or 5- year overall survival, disease-free survival and cancer-specific survival. Patients were classified into positive- or negative-myofibroblast groups according to the cut-off values that were used for stromal α-smooth muscle actin staining in each included study.

### Data extraction and collection

Two authors (Liu Liu and Lin Liu) extracted the data from the included studies using a predefined form. The following data were extracted: the name of the first author, the publication year, the country of origin of the included patients, the sample size, the type of cancer, the follow-up period(s), the endpoint(s) of interest, the definition of positively or negatively labeled myofibroblasts, and the number of patients the in positively and negatively labeled myofibroblast groups and their 3- and 5-year overall survival, disease-fress survival and cancer-specific survival. From the published studies [24,25], survival data were extracted from tables or using Kaplan-Meier curves using a digitizing software tool (Engauge Digitizer version 4.1), which converted graphs into data for both groups. One study reported the hazard ratio and its 95% confidence interval for 3-year overall survival and disease-fres survival, and we used these data directly in the subsequent combined analysis [26]. Disagreements regarding extracted data were resolved by consensus.

### Quality assessment

The quality of the included studies were independently assessed by two authors [Liu Liu and Lin Liu] using the scale described by Chen H et al [25]. The scale contained 12 items, which were categorized into the five following dimensions: patient features, ascertainment of the cancer, sample size, immunohistochemistry examination and follow-up. The scores on the scale can range from 0 to 10, with higher scores indicating better quality.

### Statistical analysis

The odds ratio and its 95% confidence interval were used to present differences in cancer prognosis between positive and negative myofibroblast groups, and *P*<0.05 was defined as indicating a significant difference. Subgroup analyses were performed by separating the data according to different types of cancers. Between-study heterogeneity was evaluated using the Q test and I^2^ test, and *P*<0.10 or I^2^ >50% was defined as suggesting the presence of between-study heterogeneity. A random-effects model was applied using the Der-Simonian and Laird method for the combined analyses because performing that analysis with a random-effects model resulted in a more conservative estimate than performing it using a fixed-effects model. Publication bias was assessed using funnel plots and Egger’s test. Visual asymmetry in a funnel plot or *P*<0.05 in an Egger’s test were defined as indicating the presence of publication bias among the included studies.

All statistical analyses were conducted using STATA 10.0 (StataCorp, College Station, TX). All statistical tests were two-sided.

## Results

### Description of the included studies

**Fig 1:**
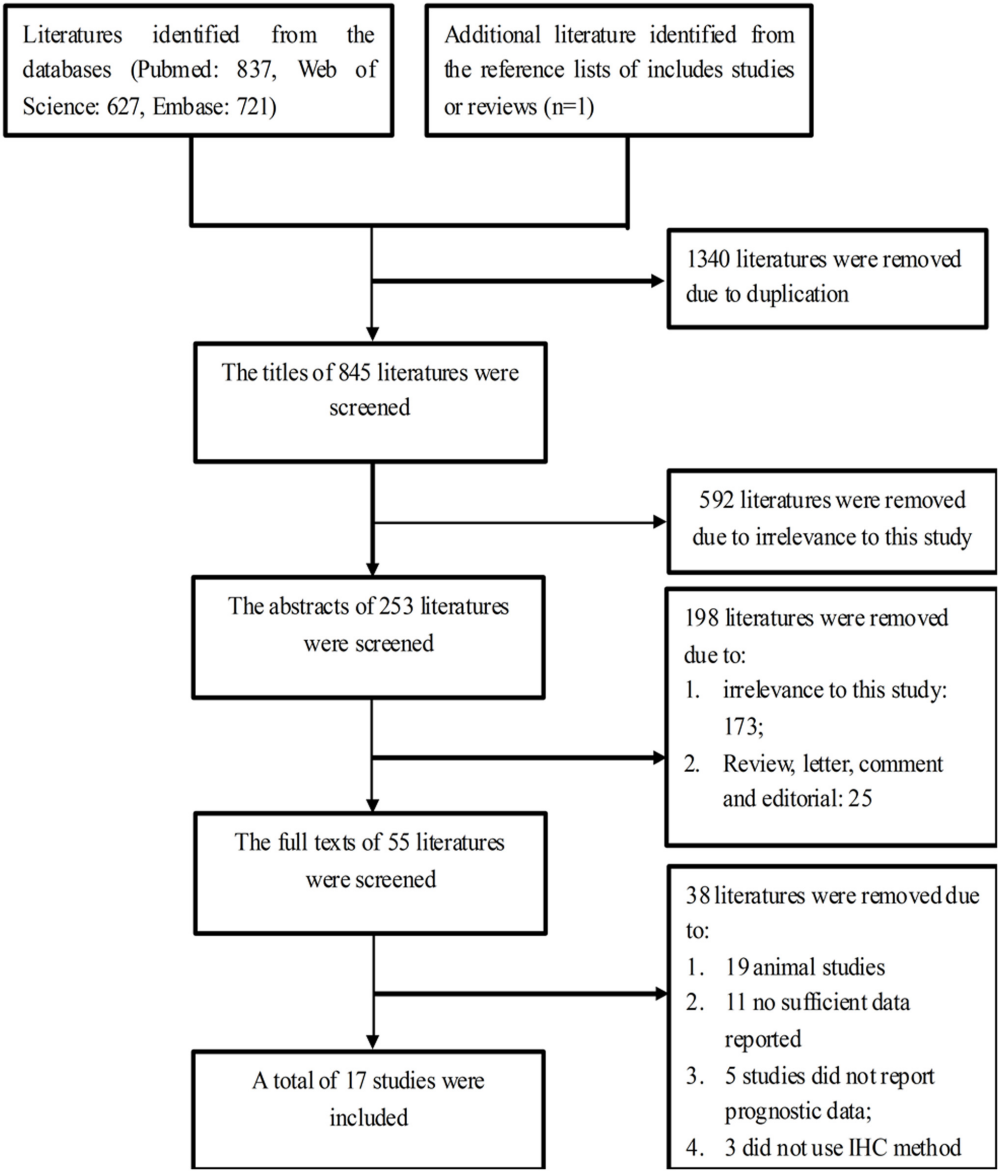
Flow chart showing the method used to select eligible studies.

Using a step-by-step selecting approach, we identified a total of 17 publications with 18 cohorts of patients for this study [11,16–22,26–34] (Fig 1). The characteristics of the included studies are shown in Tables 1 and 2, Tables A, B and C in S1 File. Five studies evaluated oral squamous cell carcinoma [11,16,19,29,32]; two studies assessed one of the following: esophageal cancer [26,28], non-small cell lung cancer [22,30], breast cancer [18,31] or liver cancer [21,27]; and four studies assessed one of the following: prostate, gastric, colorectal or pancreatic cancer [17,20,33,34]. The study published by Kilvaer T et al [22] reported data for two groups of patients: one group had non-small cell lung cancer-squamous cell carcinoma and the other group had non-small cell lung cancer-adenocarcinoma. Seven studies were conducted in European countries and the USA [11,16,18–20,22,34], and ten studies were conducted in Asian countries [17,21,26–33]. No studies were conducted in Africa.

**Table 1:** Characteristics extracted for all included studies.

	First Author [Ref.]	Publication year	Country	No. of patients	Male/ Female	Type of cancer	Follow-up (months)	TNM Stage	Endpoints of interest	Percentage of patients at 3 years (positive /negative- myofibroblast)	Percentage of patients at 5 years (positive /negative- myofibroblast)
1	Ayala G [34]	2003	USA	67	NR	Prostate cancer	Average: 46, range: 0.3–167	NR	DFS	DFS: 77.9%/55%	DFS: 72.2%44.6%
2	Surowiak P [18]	2007	Poland	45	0/45	Breast cancer	96	I-III	OS and DFS	OS: 71.5%/94.0%, DFS: 57.1%/87.9%	OS: 60.3%/94.0%, DFS: 57.2%87.9%
3	Tsujino T [17]	2007	Japan	192	NR	Colorectal cancer	NR	I-IV	DFS	DFS: 70.7%/88.6%	DFS: 58.7%/87.0%
4	Fuyuhiro Y [32]	2010	Japan	265	NR	Gastric cancer	Median: 58, range: 1–177	I-IV	OS	OS: 60.0%/81.7%	NR
5	Marsh D [16]	2011	UK	282	179/103	OSCC	Median: 76.8, range: 52.8–98.4	I-IV	CSS	CSS: 35.0%/73%	CSS: 26.0%/67.7%
6	Bello IO [11]	2011	Finland	128	60/68	OSCC	Mean: 54, range: 1–267	I-IV	CSS	CSS: 72.2%/87.9	CSS: 67.0%/87.9%
7	Yamashita M [31]	2012	Japan	60	0/60	Breast cancer	Mean: 74.8, SD: 19.3	I-III	OS and DFS	OS: 96.7%/100%, DFS: 83.8%/92.5%	OS: 84.2%/96.7%, DFS: 75.6%/92.5%
8	Fujii N [32]	2012	Japan	108	67/41	OSCC	NR	I-IV	OS	OS: 39.4%/86.0%	OS: 39.4%/86.0%
9	Wang WQ [21]	2013	China	305	255/50	Liver cancer	72	I-III	OS and DFS	OS: 68.6%/54.9%, DFS: 42.3%/59.3%	OS: 0%/54.9%, DFS: NR
10	Sinn M [20]	2014	Germany	160	NR	Pancreatic cancer	NR	I-IV	OS and DFS	OS: 24.8%44.5%, DFS: 14.5%/27.1%	OS: 16.6%37.0%, DFS: 11.7%/27.1%
11	Ding L [29]	2014	China	50	NR	OSCC	Mean: 60.34, range: 2–143	I-IV	OS	OS: 53.3%/85.0%	OS: 50.0%/85.0%
12	Ha SY [28]	2014	South Korea	116	112/4	ESCC	Median: 30, range: 0–108	I-IV	OS and DFS	OS: 50.8%/93.7%, DFS: 4501%/81.5%	OS: 41.4%/87.4%, DFS: 34.2%/75.3%
13	Chen Y [30]	2014	China	78	55/23	NSCLC	Median: 26, range: 1–55	I-IIIA	OS	OS: 31.4%/53.8%	NR
14	Parikh J [27]	2014	Japan	47	NR	Liver cancer	NR	NR	OS	OS: 10%/83.3%	NR
15	Cheng Y [26]	2015	China	95	82/13	ESCC	3 years	I-III	OS and DFS	OS: 1.87 (1.03–3.39), DFS: 1.26 (0.73–2.16)	NR
16	Luksic I [19]	2015	Croatia	152	124/28	OSCC	From 0.5 years to 5 years	NR	CSS	CSS: 77.5%/92.0%	CSS: 67.1%/92.0%
17	Kilvaer T [1] [22]	2015	Norway	255	NA	NSCLC-SCC	Median:73, range: 0–267	I-III	CSS	CSS: 72.9%/66.1%	CSS: 69.7%/63.7%
18	Kilvaer T [2] [22]	2015	Norway	201	NA	NSCLC-ADC		I-III	CSS	CSS: 66.4%/64.2%	CSS: 48.7%/47.2%

OSCC, oral squamous cell carcinoma; ESCC, esophageal squamous cell carcinoma; NSCLC-SCC, non-small cell lung cancer-squamous cell carcinoma; NSCLC-ADC, non-small cell lung cancer-adenocarcinoma; OS, overall survival; DFS, disease-free survival; CSS, cancer-specific survival, NR, not reported.

**Table 2:** Stromal α-SMA expression in all included studies.

	First Author	Publication year	Type of cancer	Cutoff for positive myofibroblasts (α-SMA staining)	Positive myofibroblast (patients, n)	Negative myofibroblast (patients, n)
1	Ayala G [34]	2003	Prostate cancer	**Positive myofibroblasts:** the expression index (EI) of α-SMA staining = 9. **Negative myofibroblasts:** EI≥0 and EI≤6; the EI of α-SMA staining was obtained by multiplying the scores for staining intensity and labeling frequency, which were determined using a 0–3 scoring system.	27/67	40/67
2	Surowiak P [18]	2007	Breast cancer	**Positive myofibroblasts:** 2–3 score for α-SMA expression. **Negative myofibroblasts:** 0–1 score for α-SMA expression. Score 1: no reaction, score 1: <10% positive myofibroblasts in the tumor stroma, score 2: 10–30% positive myofibroblasts, and score 3: >30% positive myofibroblasts.	28/45	17/45
3	Tsujino T [17]	2007	Colorectal cancer	**Positive myofibroblasts**: α-SMA expression was quantified as the percentage of the α-SMA-positive staining area out of the selected field area. Positive: >5.55% of the staining area was positive.	66/192	126/192
4	Fuyuhiro Y [32]	2010	Gastric carcinoma	The expression of α-SMA in the tumor stroma was graded as 0–4: 0, 0%; 1+, 1–24%; 2+, 25–49%; and 3+, ≥50%. **Positive myofibroblasts:** ≥2+ score. **Negative myofibroblasts:** ≤1+ score.	92/265	173/265
5	Marsh D [16]	2011	OSCC	The expression of a-SMA in the tumor stroma was scored as low/negative (<5% of the stroma was positive), moderate (patchy/focal expression, 5–50% of the stroma was positive) or high (diffuse expression throughout tumor, >50% of the stroma was positive). **Positive myofibroblasts:** high or moderate expression. **Negative myofibroblasts:** low/negative expression.	204/208	78/208
6	Bello IO [11]	2011	OSCC (tongue cancer)	Using α-SMA staining, myofibroblasts in the tumor stroma were graded as: 0, not detectable; 1, the myofibroblasts in the focal areas showed either a spindle or epithelioid morphology; 2, predominantly spindle, less dense, usually with a clear border between myofibroblasts and the tumor; 3, somewhat less dense than grade 4, or the myofibroblasts were not distributed throughout the entire tumor; 4, dense and overlapping myofibroblasts were distributed throughout the tumor and displayed a predominantly epithelioid morphology, and they showed essentially no distinct border with the tumor. Grade 0/1 was graded as **negative myofibroblasts**, and grade 2–4 was graded as **positive myofibroblasts**.	97/128	31/128
7	Yamashita M [31]	2012	Breast cancer	α-SMA expression was quantified as the relative percentage of the a-SMA staining area to that of the selected field area, with <8.48% indicating **negative myofibroblasts**. Otherwise, the sample was defined as **positive myofibroblasts**.	25/60	35/60
8	Fujii N [32]	2012	OSCC	α-SMA expression was graded as: 0, negative; 1, a small number of scattered cells were stained; 2, irregular and non-continuous focal staining; and 3: abundant staining. **Positive myofibroblasts**: grade 2 and 3, and **negative myofibroblasts**: grade 0–1.	33/108	75/108
9	Wang WQ [21]	2013	Liver cancer	**Positive or negative myofibroblasts** was defined according to α-SMA expression: the positive staining area/total area, with the median value used as the cutoff.	153/305	152/305
10	Sinn M [20]	2014	Pancreatic cancer	Staining intensity was defined as negative, weak, moderate or strong. Negative and weak staining were defined as **negative fibroblasts**, and moderate and strong were defined as **positive myofibroblasts**.	133/160	27/160
11	Ding L [29]	2014	OSCC	α-SMA expression was classified using 4 grades (grade 0: negative for α-SMA expression, grade 3: the highest level of α-SMA expression). Grades 0/1 were defined as **negative myofibroblasts**, while grades 2 and 3 were defined as **positive fibroblasts**.	30/50	20/50
12	Ha SY [28]	2014	ESCC	α-SMA expression was graded as: 1, weak staining in 50% or moderate staining in 20% of the tumor stroma; 2, weak staining in 50%, moderate staining in 20–50% or strong staining in 20% of the tumor stroma; and 3, moderate staining in 50% or strong staining in 20% of the tumor stroma. **Positive myofibroblasts**: a score of 2 or 3; otherwise, the sample was regarded as negative.	96/116	20/116
13	Chen Y [30]	2014	NSCLC	The staining intensity and labeling frequency of α-SMA were determined using a 0–3 scoring system and a 0–2 scoring system, respectively. The expression index for a-SMA was obtained by multiplying the scores for staining intensity and labeling frequency. **Positive myofibroblasts**: a score higher than 2, and **negative myofibroblasts**: a score between 0 and 2.	22/78	56/78
14	Parikh J [27]	2014	Liver cancer	**Positive myofibroblasts**: grade 2–3 for α-SMA staining in the liver, and **negative myofibroblasts**: grade 0–1 for α-SMA staining. α-SMA staining: 0, no staining; +1, staining intensity considerably lower than that in vascular smooth muscle cells (VSMCs); +2, staining intensity lower than VSMCs; and +3, staining intensity similar to VSMCs.	41/47	6/47
15	Cheng Y [26]	2015	ESCC	α-SMA-rich (**positive myofibroblasts**) indicated dense overlap in the staining, which was distributed throughout the tumor, predominantly in cells with an epithelioid morphology and showing essentially no distinct border with the ESCC. In contrast, a-SMA-poor (**negative myofibroblasts**) indicated somewhat less dense staining, or the staining was not distributed throughout the entire tumor.	49/95	46/95
16	Luksic I [19]	2015	OSCC	Scoring for α-SMA expression: 0: no staining, 1: 1% to 25% of the stroma was stained, 2: 26–50% of the stroma was stained, 3: 51–75% of the stroma was stained, and 4: more than 76% of the stroma was stained. **Negative myofibroblasts:** grade 0 and 1 staining, and **positive myofibroblasts**: grade 2–4 staining.	110/152	42/152
17	Kilvaer T [1] [22]	2015	NSCLC-SCC	Scoring system for α-SMA expression: 0: no staining, 1: 1–10%, 2: 11–50% and 3: > 50% of the stroma was stained. Grade 0 and 1 were both regarded as **negative myofibroblasts**, and grade 2–3 were regarded as **positive myofibroblasts**.	57/255	198/2555
18	Kilvaer T [2] [22]	2015	NSCLC-ADC		64/201	137/201

OSCC, oral squamous cell carcinoma; ESCC, esophageal squamous cell carcinoma; NSCLC-SCC, non-small cell lung cancer-squamous cell carcinoma; NSCLC-ADC, non-small cell lung cancer-adenocarcinoma; α-SMA, α-smooth muscle actin.

A total of 2606 patients with solid cancers were included, and the sample sizes of the included studies ranged from 45 to 305 [17,21]. Eleven studies containing 1329 patients reported overall survival [18,20,21,26–33], eight studies containing 1040 patients reported disease-free survival [17,18,20,21,26,28,31,34], and four studies containing 987 patients reported cancer specific survival [11,16,19,22].

The qualities of the included studies were evaluated (Table D in S1 File). Ten studies were rewarded a score of 6 or more [11,16,18,21,22,28–31,33], indicating that they were of high quality. In detail, 7 of the studies used a negative or positive control for immunohistochemistry staining [17,18,21,22,28,29,32], and nine studies reported that a staining scores for α-smooth muscle actin in stromal myofibroblasts were independently assessed by two individuals [11,16,18,20,22,28–30,33]. In addition, twelve studies described follow-up data for the patients [11,16,18,21,22,26,28–31,33,34].

All of the included studies used α-smooth muscle actin as a specific marker for myofibroblasts in the tumor stroma. The cutoff values for the positive myofibroblast patients depended on the immunohistochemistry staining score and the method that was used in each included study (Table 2). In addition, three studies divided the samples according to their staining intensity for α-smooth muscle actin into low, medium and rich groups [11,16,32]. We therefore grouped the medium and rich levels together into a “positive myofibroblast group”, whereas a low level of α-smooth muscle actin staining defined the “negative myofibroblast group”.

### Impact of stromal myofibroblasts on overall survival

**Fig 2:**
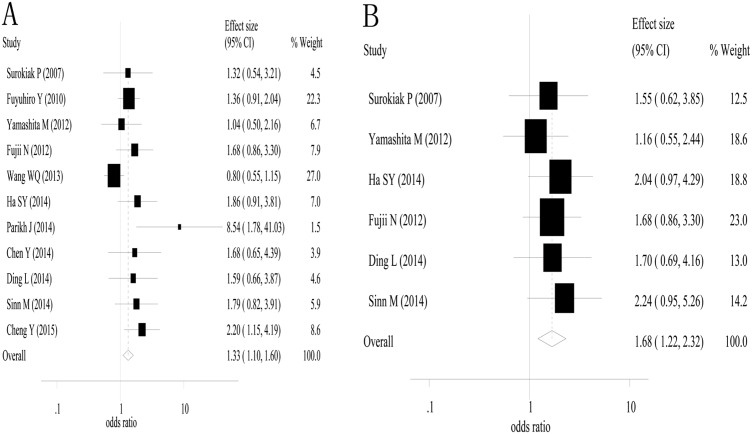
A high density of α-smooth muscle actin-labeled stromal myofibroblasts was associates with poor 3- and 5-year overall survival. A, 3-year overall survival; and B, 5-year overall survival.

The combined analysis of 3-year overall survival was conducted based on eleven studies. The combined odds ratio and its 95% confidence interval were 1.33 (1.10–1.60) with *P*<0.01 for the positive myofibroblast versus negative myofibroblast group (Fig 2a) [18,20,21,26–33]. Between-study heterogeneity was not found (I^2^ = 44.5% and the *P* value for Q test = 0.055). Six studies [18,20,28,29,31,32] were used in the combined analysis of 5-year overall survival, and the estimated odds ratio and its 95% confidence interval were 1.68 (1.22–2.32) with *P*<0.01 (Fig 2b). We did not observe heterogeneity between the included studies (I^2^ = 0% and the *P* value in the Q test = 0.89). These results suggested that α-smooth muscle actin-labeled stromal myofibroblasts were associated with poor 3- and 5-year overall survival in solid cancers.

Subgroup analyses of 3-year overall were performed for breast cancer (2 studies), oral squamous cell carcinoma (2 studies), liver cancer (2 studies) and esophageal squamous cell carcinoma (2 studies). The estimated odds ratios and their 95%confidence intervals were 1.149 (0.65–2.01) for breast cancer (*P* = 0.64), 1.65 (0.97–2.82) for oral squamous cell carcinoma (*P* = 0.07), 2.29 (0.23–23.17) for liver cancer (*P* = 0.48) and 2.04 (1.26–3.29) for esophageal squamous cell carcinoma (*P*<0.01). A subgroup analyses for 5-year overall survival was also performed for breast cancer (2 studies) and oral squamous cell carcinoma (2 studies). The estimated odds ratios and their 95% confidence intervals were 1.30 (0.73–2.32) for breast cancer (*P* = 0.37) and 1.69 (0.99–2.89) for oral squamous cell carcinoma (*P* = 0.06).

### The impact of stromal myofibroblasts on disease-free survival

**Fig 3:**
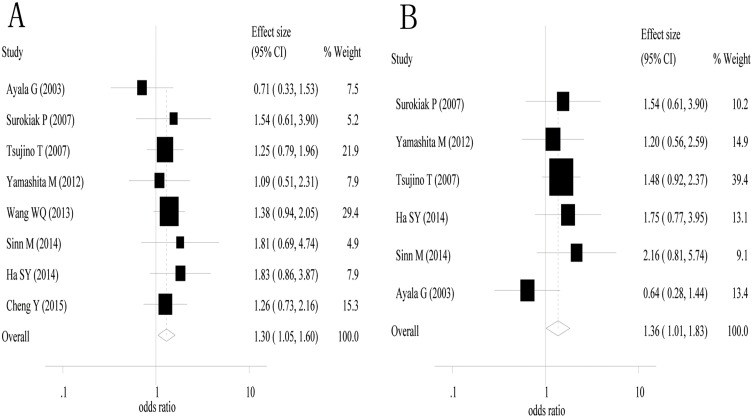
A high density of α-smooth muscle actin-labeled stromal myofibroblasts was associated with poor 3- and 5-year disease-free survival. A, 3-year disease-free survival; and B, 5-year disease-free survival.

Eight studies [17,18,20,21,26,28,31,34] were used in the combined analysis of 3-year disease-free survival. The estimated odd ratios and its 95%confidence interval were 1.30 (1.05–1.60) with *P* = 0.016 (Fig 3a). There was no heterogeneity among the included studies (I^2^ = 0% and *P* value for the Q test = 0.764). Six studies [17,18,20,28,31,34] were used in the combined analysis of 5-year disease free survival. The estimated odds ratio and its 95%confidence intervals were 1.36 (1.01–1.83) with *P* = 0.041 (Fig 3b). There was no heterogeneity among the included studies (I^2^ = 0% and *P* value for the Q test = 0.436). These results suggest that α-smooth muscle actin-labeled stromal myofibroblasts are associated with poor 3- and 5-year disease-free survival in patients with solid cancers.

A subgroup analyses was performed for 3-year disease-free survival for breast cancer (2 studies) and esophageal squamous cell carcinoma (2 studies). The estimated odors ratio and 95%confidence intervals were 1.25 (0.70–2.24) for breast cancer (*P* = 0.453) and 1.43 (0.92–2.22) for esophageal squamous cell carcinoma (P = 0.109). Subgroup analyses were performed for 5-year disease-free survival for breast cancer (2 studies), and the estimated odds ratio and its 95%confidence interval was 1.33 (0.74–2.40) with P = 0.342.

### The impact of stromal myofibroblasts on cancer-specific survival

**Fig 4:**
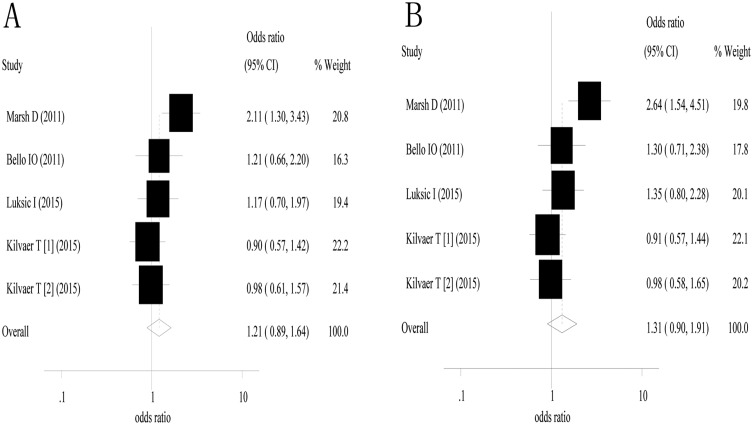
α-smooth muscle actin-labeled stromal myofibroblasts were not associated with cancer-specific survival. A, 3-year cancer-specific survival; and B, 5-year cancer-specific survival.

Four studies containing five groups of patients [11,16,19,22] were included in the analysis of 3- and 5-year cancer-specific survival. The estimated odds ratio and 95% confidence interval for 3-year cancer-specific survival were 1.21 (0.89–1.64) with *P* = 0.229 (Fig 4a). There was no obvious heterogeneity among the included studies (I^2^ = 46.4% and *P* value for the Q test = 0.113). The estimated odds ratio and 95% confidence interval for 5-year cancer-specific survival were 1.31 (0.90–1.91) with *P* = 0.155 (Fig 4b). Between-study heterogeneity was found between the included studies (I^2^ = 60.8% and *P* value for the Q test = 0.037).

A subgroup analysis was conducted for oral squamous cell carcinoma (three studies). The estimated odds ratio and 95% confidence interval for 3-year CSS were 1.47 (0.99–2.18) with *P* = 0.054, and the odds ratio and 95% confidence interval for 5-year cancer-specific survival were 1.68 (1.06–2.65) with *P* = 0.027.

### Assessment of publication bias

**Fig 5:**
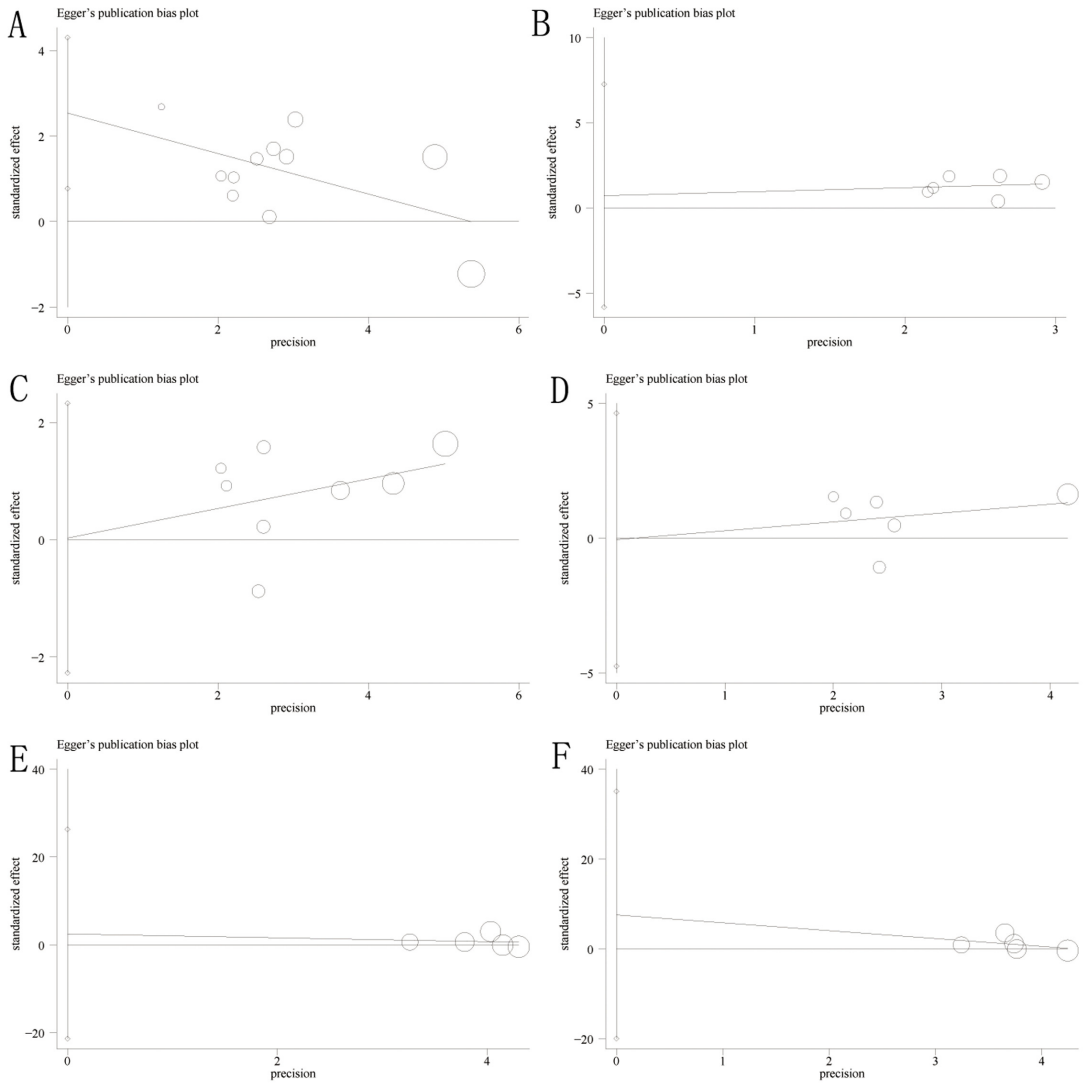
The Egger’s publication bias plot suggested that there was no publication bias for overall survival, disease-free survival and cancer-specific survival. A-B, 3- and 5-year overall survival; C-D, 3- and 5-year disease-free survival; and E-F, 3- and 5-year cancer-specific survival.

Publication bias was assessed for overall survival, disease-free survival and cancer-specific survival. Using funnel plots and Egger’s tests, we found that there was no significant publication bias in the analyses of overall survival, disease-free survival and cancer-specific survival (*P* = 0.09 and 0.818 for Egger’s tests of 3- and 5-year overall survival, respectively; *P* = 0.413 and 0.626 for Egger’s test of 3- and 5-year disease-free survival, respectively; and *P* = 0.832 and 0.503 for Egger’s test of 3- and 5-year cancer-specific survival, respectively) (Fig 5A–5F).

## Discussion

The TNM staging system is based on the biological features of cancer cells and is used as a foundation for categorizing patients with solid cancers into those with early and advanced stage cancer, which is then used to determine the most reasonable treatment strategy. However, the TNM system is inadequate because patients with same TNM stage can have different prognoses. Introducing a parameter that incorporates information about the tumor microenvironment would significantly supplement the TNM staging system and would be helpful when determining personalized treatment strategies in these patients. To our knowledge, this is the first study to perform a meta-analysis evaluating the association between α-smooth muscle actin-labeled stromal myofibroblasts and prognoses in solid cancers. The results of this study suggest that the abundant presence of stromal myofibroblasts in the cancer stroma is associated not only with poor overall survival but also with unfavorable disease-free survival.

Cancer-associated fibroblasts have a large, plump, and spindle-shaped morphology. Similar to mesenchymal stem cells, cancer-associated fibroblasts have a remarkable capacity to transdifferentiate into cartilage cells and bone cells [35]. Cancer-associated fibroblasts are activated by an increase in the expression of α-smooth muscle actin, which causes them to transdifferentiate into myofibroblasts when they are exposed to inflammatory cytokines from cancer cells [13,36]. Moreover, cancer-associated fibroblasts secrete a number of growth factors and inflammatory chemokines that stimulate proliferation in cancer cells, enhance angiogenesis and epithelial-mesenchymal transition, and eventually accelerate cancer growth in addition to local and distant metastasis [36,37]. Myofibroblasts in the cancer stroma are regarded as a subgroup of cancer-associated fibroblasts [13]. Many experimental studies have suggested that similar to cancer-associated fibroblasts, myofibroblasts promote cancer progression and metastasis by expressing high levels of inflammatory factors and chemokines, such as interleukin-6 and C-X-C motif chemokine [38,39].

An increasing amount of evidence has shown that stromal myofibroblasts promote cancer progression, and this has pushed researchers to investigate whether myofibroblasts can be used as a prognostic marker for solid cancers. Moreover, if stromal myofibroblasts are a main component of the tumor stroma, are they a potential target for cancer treatments? To answer this question, many clinical studies have been conducted. However, no conclusive answer has yet been reached. Ha SY et al [28] assessed α-smooth muscle actin expression in stromal myofibroblasts in 116 cases of esophageal squamous cell carcinoma, and their results suggested that stromal α-smooth muscle actin was expressed at higher levels in larger esophageal squamous cell carcinomas and in advanced T-stage and N-stage esophageal squamous cell carcinomas. In addition, esophageal squamous cell carcinoma patients with higher expression levels of stromal α-smooth muscle actin had lower 5-year overall survival and disease-free survival than patients with lower α-smooth muscle actin expression [28]. Similarly, some studies have reported that stromal fibroblasts are associated with a high risk of recurrence and poor prognosis in other types of solid cancers, such as breast, colorectal and gastric cancer [17,18,33]. Furthermore, targeting stromal myofibroblasts suppressed growth in cholangiocarcinomas and improved host survival in an experimental study [12]. Our study evaluated the impact of stromal myofibroblasts on prognoses in solid cancers. The results suggest that stromal myofibroblasts lead not only to poor overall survival but also to unfavorable disease-free survival. In addition, the results of a stratified analysis also suggested that a high density of stromal myofibroblasts is associated with shorter cancer-specific survial in OSCC. Although the impact of stromal myofibroblasts on poor survival in solid cancers has been described in many studies, some authors do not support the existence of this relationship. Ayala et al reported that in prostate cancer, a low density of stromal myofibroblasts was more correlated with shorter disease-free survival than a high density of cancer-associated fibroblasts [34]. Similarly, Wang WQ showed that α-smooth muscle actin-labeled cancer-associated fibroblasts were not associated with overall survival or disease-free survival in either hepatic or pancreatic cancer [21]. These inconsistent results might be caused by differences in research methods or the number of patients included in the study. Therefore, studies including larger sample size are needed in the future.

This study is meaningful because our data suggest that stromal myofibroblasts are an effective marker for predicting prognoses in patients with solid cancers. Moreover, unlike current chemotherapies that target tumor cells, a therapy that targets myofibroblasts would be a novel avenue for research in cancer therapies in the future. We should mention that there were some limitations to this study. First, the included studies were retrospective, which means they were susceptible to some bias. Second, the sample sizes of the included studies were relatively small. Finally, there was heterogeneity among the included, and this might impair the accuracy of their pooled estimates. To overcome these shortages, we used a random-effects model rather than a fixed-effects model because a random-effect model is more conservative for a combined analyses.

In summary, this study suggests that a high density of stromal myofibroblasts, which were identified using α-smooth muscle actin as a marker, contributed to poor survival in patients with solid cancers. These data could therefore be used to identify high-risk patients who may need more intense therapy.

## Supporting Information

S1 File Table A-C The characteristics of the included studies; Table D Quality assessment of all included studies.
(DOCX)

S1 PRISMA Checklist PRISMA checklist. (DOC)
