# Supplementary material for: Stromal Myofibroblasts Are Associated with Poor Prognosis in Solid Cancers: A Meta-Analysis of Published Studies
Source: PLoS One. 2016 Jul 26;11(7):e0159947. doi: 10.1371/journal.pone.0159947 (PMC4961396; doi:10.1371/journal.pone.0159947)
Supplement: S1 File — Table A-C The characteristics of the included studies; Table D Quality assessment of all included studies. (DOCX) [file pone.0159947.s001.docx]

**Supplementary table A Overall survival (OS) of patients in positive- or negative-myofibroblast groups**

| **Author** | **Year** | **Country** | **Sample size** | **Cancer type** | **3-year OS/total patients**  **(positive myofibroblast, n)** | **3-year OS/total patients**  **(negative myofibroblast, n)** |  | **5-year OS /total patients**  **(positive myofibroblast, n)** | **5-year OS/total patients**  **(negative myofibroblast, n)** |
| --- | --- | --- | --- | --- | --- | --- | --- | --- | --- |
| Surokiak P | 2007 | Poland | 45 | Breast cancer | 20/28 | 16/17 |  | 17/28 | 16/17 |
| Fuyuhiro Y | 2010 | Japan | 265 | Gastric cancer | 55/92 | 141/173 |  | NR | NR |
| Yamashita M | 2012 | Japan | 60 | Breast cancer | 24/25 | 35/35 |  | 21/25 | 34/35 |
| Fujii N | 2012 | Japan | 108 | OSCC | 17/33 | 65/75 |  | 17/33 | 65/75 |
| Wang WQ | 2013 | China | 305 | Liver cancer | 105/153 | 83/152 |  | NR | NR |
| Sinn M | 2014 | Germany | 160 | Pancreatic cancer | 33/133 | 12/27 |  | 22/133 | 10/20 |
| Ding L | 2014 | China | 50 | OSCC | 16/30 | 17/20 |  | 15/30 | 17/20 |
| Ha SY | 2014 | South Korea | 116 | ESCC | 49/96 | 19/20 |  | 40/96 | 17/20 |
| Chen Y | 2014 | China | 78 | NSCLC | 7/22 | 30/56 |  | NR | NR |
| Parikh J | 2014 | Japan | 47 | Liver cancer | 4/41 | 5/6 |  | NR | NR |
| Cheng Y^¶^ | 2015 | China | 95 | ESCC | NR^¶^ | NR^¶^ |  | NR | NR |

¶ Hazard ratio (HR) and 95% confidence interval (95%CI), rather than number of patients, for 3-year OS was reported and used for combined analysis;

OSCC, oral squamous cell carcinoma; ESCC, esophageal squamous cell carcinoma; NSCLC, non-small cell lung cance; OS, overall survival; NR, not reported.

**Supplementary table B Disease-free survival (DFS)of patients in positive- or negative-fibroblast groups**

| **Author** | **Year** | **Country** | **Sample size** | **Cancer** | **3-year DFS/total patients (positive myofibroblast, n)** | **3-year DFS/total patients (negative myofibroblast, n)** |  | **5-year DFS/total patients (positive myofibroblast, n)** | **5-year DFS/total patients (negative myofibroblast, n)** |
| --- | --- | --- | --- | --- | --- | --- | --- | --- | --- |
| Ayala G | 2003 | USA | 67 | Prostate cancer | 21/27 | 22/40 |  | 19/27 | 18/20 |
| Surokiak P | 2007 | Poland | 45 | Breast cancer | 16/28 | 15/17 |  | 16/28 | 15/17 |
| Tsujino T | 2007 | Japan | 192 | Colorectal cancer | 47/66 | 112126 |  | 39/66 | 110/126 |
| Yamashita M | 2012 | Japan | 60 | Breast cancer | 21/25 | 32/35 |  | 19/25 | 32/35 |
| Wang WQ | 2013 | China | 305 | Liver cancer | 65/153 | 90/152 |  | NR | NR |
| Sinn M | 2014 | Germany | 160 | Pancreatic cancer | 19/133 | 7/27 |  | 16/133 | 7/27 |
| Ha SY | 2014 | South Korea | 116 | ESCC | 43/96 | 16/20 |  | 33/96 | 15/20 |
| Cheng Y | 2015 | China | 95 | ESCC | NR^¶^ | NR^¶^ |  | NR | NR |

¶ Hazard ratio (HR) and 95% confidence interval (95%CI), rather than number of patients, for 3-year DFS was reported and used for combined analysis;

OSCC, oral squamous cell carcinoma; ESCC, esophageal squamous cell carcinoma; NSCLC, non-small cell lung cancer; DFS, disease free survival; NR, not reported.

**Supplementary table C Cancer-specific survival (CSS) of patients in positive- or negative-myofibroblast groups**

| **Author** | **Year** | **Country** | **Sample size** | **Cancer** | **3-year CSS/death**  **(positive myofibroblast, n)** | **3-year CSS/death (negative myofibroblast, n)** |  | **5-year CSS/death**  **(positive myofibroblast, n)** | **5-year CSS/death (negative myofibroblast, n)** |
| --- | --- | --- | --- | --- | --- | --- | --- | --- | --- |
| Marsh D | 2011 | UK | 208 | OSCC | 27/78 | 149/204 |  | 20/78 | 138/204 |
| Bello IO | 2011 | Finland | 128 | OSCC | 70/97 | 27/31 |  | 65/97 | 27/31 |
| Luksic I | 2015 | Croatia | 152 | OSCC | 85/110 | 39/43 |  | 74/110 | 39/43 |
| Kilvaer T [1] | 2015 | Norway | 288 | NSCLC-SCC | 42/57 | 131/198 |  | 40/57 | 126/198 |
| Kilvaer T [2] | 2015 | Norway | 211 | NSCLC-ADC | 42/64 | 88/137 |  | 31/64 | 65/137 |

OSCC, oral squamous cell carcinoma; ESCC, esophageal squamous cell carcinoma; NSCLC-SCC, non-small cell lung cancer- squamous cell carcinoma; NSCLC-ADC, non-small cell lung cancer-adenocarcinoma; CSS, cancer-specific survival.

**Supplementary table D Quality assessment of all included studies.**

| **Items** | Ayala G | Surokiak P | Tsujino T | Fuyuhiro Y | Marsh D | Bello IO | Yamashita M | Fujii N | Wang WQ | Sinn M | Ding L | Ha SY | Chen Y | Parikh J | Cheng Y | Luksic I | Kilvaer T |
| --- | --- | --- | --- | --- | --- | --- | --- | --- | --- | --- | --- | --- | --- | --- | --- | --- | --- |
| **Representativeness of cases** |  |  |  |  |  |  |  |  |  |  |  |  |  |  |  |  |  |
| 1. Consecutive or random recruitment from case population (1 score) | 1 | 1 | 1 | 1 | 1 | 1 | 1 |  | 1 | 1 | 1 | 1 | 1 | 1 | 1 | 1 | 1 |
| 1. No method of selection stated (0 score) |  |  |  |  |  |  |  | 0 |  |  |  |  |  |  |  |  |  |
| **Ascertainment of cancer** |  |  |  |  |  |  |  |  |  |  |  |  |  |  |  |  |  |
| 1. Histological confirmation at the department of pathology (2 scores) | 2 | 2 | 2 | 2 | 2 | 2 | 2 | 2 | 2 | 2 | 2 | 2 | 2 | 2 | 2 | 2 | 2 |
| 1. Medical record (1score) |  | 1 | 1 | 1 | 1 |  | 1 | 1 | 1 | 1 | 1 |  | 1 | 0 | 1 | 1 | 1 |
| 1. Not described (0 score) |  |  |  |  |  |  |  |  |  |  |  |  |  |  |  |  |  |
| **Sample size** |  |  |  |  |  |  |  |  |  |  |  |  |  |  |  |  |  |
| 1. >=200 (1 score) |  |  |  | 1 | 1 |  |  |  | 1 |  |  |  |  |  |  |  | 1 |
| 1. <200 (0 score) | 0 | 0 | 0 |  |  | 0 | 0 | 0 |  | 0 | 0 | 0 | 0 | 0 | 0 | 0 |  |
| **Immunohistochemistry examination** |  |  |  |  |  |  |  |  |  |  |  |  |  |  |  |  |  |
| 1. Negative or positive control (1 score) |  | 1 | 1 |  |  |  |  | 1 | 1 |  | 1 | 1 |  |  |  |  | 1 |
| 1. Diagnosed by two experienced pathologists (1 score) |  | 1 |  | 1 | 1 | 1 |  |  |  | 1 | 1 | 1 | 1 |  |  |  | 1 |
| 1. Diagnosed under “blinded” condition (1 score) |  |  |  | 1 |  | 1 |  |  |  |  |  | 1 |  |  |  |  |  |
| **Follow-up of patients** |  |  |  |  |  |  |  |  |  |  |  |  |  |  |  |  |  |
| 1. Describe follow-up time (1 score) | 1 | 1 |  | 1 | 1 | 1 | 1 |  | 1 |  | 1 | 1 | 1 |  | 1 |  | 1 |
| 1. Describe how many patients were lost to follow up or not available for statistical analysis (1 score) |  | 1 |  | 1 | 1 |  | 1 |  | 1 |  | 1 |  | 1 |  |  |  | 1 |
| **Total scores** | 4 | 7 | 5 | 9 | 8 | 6 | 6 | 4 | 8 | 5 | 8 | 7 | 7 | 3 | 5 | 4 | 9 |
|  |  |  |  |  |  |  |  |  |  |  |  |  |  |  |  |  |  |
